# Construction Notes

**Published:** 1893-03-04

**Authors:** 


### CONSTRUCTION NOTES.

The alterations at Grantham General Hospital have now been completed; the kitchen and the Matron's rooms having been extended from designs of Mr. Adolphus Carne, of London, and the building effected at a cost of £550 by Mr. John Shebourn, of How-on-the-Hill.

The new Thornton and District Joint Hospital for Infectious Diseases was designed by Messrs. W. and Y. B. Bailey, of Bradford and Keighley, architects. The building has been arranged with a view to the complete isolation of its several apartments. There are four wards for six beds and six cots, and two nurses' kitchens. The administrative block contains living-rooms for the master and matron, and bed-rooms, a sitting-room for the nurses, a doctor's-room, and an ambulance-room. There is also a detached block of buildings containing wash-house, laundry, mortuary, and disinfecting-rooms. The latter contain a steam disinfecting apparatus made by Messrs. Goddard, Massey, and Warner, of Nottingham. The total cost of the building, including fitting and furnishing and site of three acres, has been about £3,000.

The public opening of the new hospital at Scarborough is fixed for February 27th, at which H.R.H. Princess Christian is most probably to be asked to preside. The plans for the building were designed by Messrs. Hall and Tugwoll, architects, the builder, Mr. John Barry, securing the contract at £5,800. The principal wards are on the first floor, airy light rooms some 30 feet square. The main wards are to be divided by a partition. There are a series of small wards for private paying patients, besides day-room, children's and convalescent wards. At the end of each wing is a ventilation chamber, large rooms into which the ends of the ventilation shafts are brought, and a special apparatus for exhausting the foul air from every room in the building. There will eventually be a hydraulic lift, the size of an ordinary bed, for the conveyance of patients in a vertical position. This is already arranged for at the main entrance of the building.

Tenders have been accepted for the completion of the new buildings at the Great Northern Central Hospital, Holloway Road, at the specified sum of £25,000, exclusive of furnishing, &c. Towards this amount £8,000 only have been received. The proposed extension will increase the number of beds to 150, out of which it is intended to set aside a few for those patients who require operations and treatment afterwards, such as they cannot receive at their own homes, and who are able to contribute some small sum towards the expense incurred. The Baroness Burdett-Coutts has already raised £5,000 for the spacious out-patient department, and the Ladies' Association, over which the Baroness presides, have expressed their intention of raising another £5,000 to provide a women's ward in the new wing, which is to be erected as a memorial to the Duke of Clarence. This extension will contain rectangular and circular wards. The same amount of cubicle space is allotted each bed, as in the ordinary straight wards.

